# Long-Term Follow-Up of Patients Receiving Neoadjuvant Treatment Modalties for Soft Tissue Sarcomas of the Extremities

**DOI:** 10.3390/cancers13205244

**Published:** 2021-10-19

**Authors:** Miriam Rauch, Abbas Agaimy, Sabine Semrau, Alexander Willner, Oliver Ott, Rainer Fietkau, Werner Hohenberger, Roland S. Croner, Robert Grützmann, Katja Fechner, Nikolaos Vassos

**Affiliations:** 1 Department of Surgery, University Hospital Erlangen, Friedrich-Alexander-University Erlangen-Nürnberg (FAU), 91054 Erlangen, Germany; miriam.rauch@gmail.com (M.R.); werner.hohenberger@uk-erlangen.de (W.H.); robert.gruetzmann@uk-erlangen.de (R.G.); katja.fechner@uk-erlangen.de (K.F.); 2 Faculty of Medicine, Friedrich-Alexander-University Erlangen-Nürnberg (FAU), 91054 Erlangen, Germany; 3 Department of Pathology, University Hospital Erlangen, Friedrich-Alexander-University Erlangen-Nürnberg (FAU), 91054 Erlangen, Germany; abbas.agaimy@uk-erlangen.de; 4 Department of Radiation Oncology, University Hospital Erlangen, Friedrich-Alexander-University Erlangen-Nürnberg (FAU), 91054 Erlangen, Germany; sabine.semrau@uk-erlangen.de (S.S.); alexander.willner@uk-erlangen.de (A.W.); oliver.ott@uk-erlangen.de (O.O.); rainer.fietkau@uk-erlangen.de (R.F.); 5 Department of Surgery, University Hospital Magdeburg, 39106 Magdeburg, Germany; roland.croner@med.ovgu.de; 6 Mannheim University Medical Center, Division of Surgical Oncology, Department of Surgery, University of Heidelberg, 68167 Mannheim, Germany

**Keywords:** soft tissue sarcoma, extremity, neoadjuvant therapy, radiochemotherapy, radiation

## Abstract

**Simple Summary:**

Neoadjuvant radiotherapy has gained popularity as a treatment strategy for locally advanced soft tissue sarcoma of the extremities. However, little is yet known about the benefits and risks of adding preoperative chemotherapy. High-risk patients with soft tissue sarcomas of the extremities are treated with a combined preoperative radiochemotherapy at our institution. This study reports an analysis of patients treated either with primary surgery or with preoperative radiochemotherapy, followed by surgery. This kind of multimodal therapy leads to excellent oncological outcomes and is associated with low rates of severe postoperative complications in highly specialized centers.

**Abstract:**

Background: Neoadjuvant treatment modalities in soft tissue sarcoma (STS) of the extremities have become more popular in recent years, but because of the rarity and heterogeneity of STS, there are yet few studies on the long-term impact of neoadjuvant treatment modalities, especially in terms of neoadjuvant radiochemotherapy. Methods: The study enrolled 136 patients with primary STS of the extremities who underwent surgery with curative intent or neoadjuvant therapy, followed by surgery in a 15-year period. Neoadjuvant treatment consisted of radiotherapy (RT) with 60 Gy and in most cases simultaneous chemotherapy (CTx) with ifosfamide (1.5 g/m^2^/d, d1–5, q28) and doxorubicine (50 mg/m^2^/d, d3, q28). We investigated the clinical, (post)-operative and histopathological data and the oncological follow-up as well. The median follow-up period was 82 months (range 6–202). Results: A total of 136 patients (M:F = 73:63) with a mean age of 62 years (range; 21–93) was observed. Seventy-four patients (54.4%) received neoadjuvant therapy (NT), 62 patients (45.6%) received primary surgery (PS). When receiving NT, patients with high-risk STS had a lower risk to develop distant metastasis (*p* = 0.025). Age, histological type, tumor size and surgical margins (R0 vs. R1) had no influence on any survival rates. There was an association between NT and the occurrence of postoperative complications (*p* = 0.001). The 5-year local recurrence free survival (LRFS), metastasis free survival (MFS), disease free survival (DFS) and overall survival (OS) rate of the whole cohort was 89.9%, 77.0%, 70.6% and 72.6%; whereas the 5-year LRFS, MFS, DFS and OS rate was 90.5%, 67.2%, 64.1% and 62.8% for the NT group and 89.5%, 88.3%. 78.4% and 83.8% for the PS group. Conclusions: Multimodal treatment strategies in patients with STS of extremities lead to excellent oncological outcomes. Patients with high-risk STS had a significantly better MFS when receiving NT than patients with low-risk STS. NT was associated with a higher probability of postoperative but well-manageable complications.

## 1. Introduction

Although neoadjuvant treatment modalities of STS of the extremities have become more and more popular over the last decade [1,2], little is known about the advantages of a combined radiochemotherapeutic approach. There have been few studies focused on this interaction to date. After the first steps into combined radiochemotherapy (RCT) had been taken [3,4,5,6,7,8,9,10], it became evident that combining RT with CTx seemed to be a promising therapeutic approach for high-risk STS. Studies from DeLaney et al. [11], Mullen et al. [12] and Kraybill et al. [13,14], where a combination of RT with CTx (MAID regimen; combination of mesna, doxorubicin, ifosfamide and dacarbazine) was performed, are typical examples of this therapeutic approach. The retrospective database analysis by Mahmoud et al. [15] is of particular interest, as it is the largest study (*n* = 3422) of the effect of adjunct therapy on high-risk patients with STS of the extremities to date, showing that a neoadjuvant combination of radiotherapy and chemotherapy (nRCT) may be better than surgery only and better than RT or CTx alone.

At our institution, nRCT was applied to patients with unfavorable conditions for a complete resection of STS already in 1992 as a phase-II-study [16]. These conditions included: unresectable or borderline resectable STS, unfavorable tumor location (retroperitoneum or head and neck region, infiltration of vessels and bones), high-risk tumor (tumor size ≥ 5 cm, grade II-III, deep/extracompartmental location) and recurrent sarcoma. This approach was proved to be a safe and effective treatment providing good 3-year survival rates (OS, MFS and DFS: 83%, 68% and 64%, respectively) [16]. Furthermore, a recent analysis of advanced STS at our institution confirmed that neoadjuvant radiochemotherapy is associated with good feasibility and high local efficacy (5-year OS, LRFS, MFS and DFS survival rate of 83.3%, 89.9% and 66.6%, respectively) [16,17].

The aim of this study is the evaluation of the data collected for over 15 years at our institution and the analysis of the impact of neoadjuvant radio(chemo)therapy followed by surgery compared with primary surgery on the survival of different patient groups. Additionally, since neoadjuvant therapy is associated with increased risk of postoperative complications [18,19,20,21,22,23,24], it would be important to assess if the benefits of neoadjuvant treatment modalities outweigh the possible disadvantages of having increased risk of postoperative morbidity.

## 2. Patients and Methods

### 2.1. Patient Selection and Data

**Figure 1:**
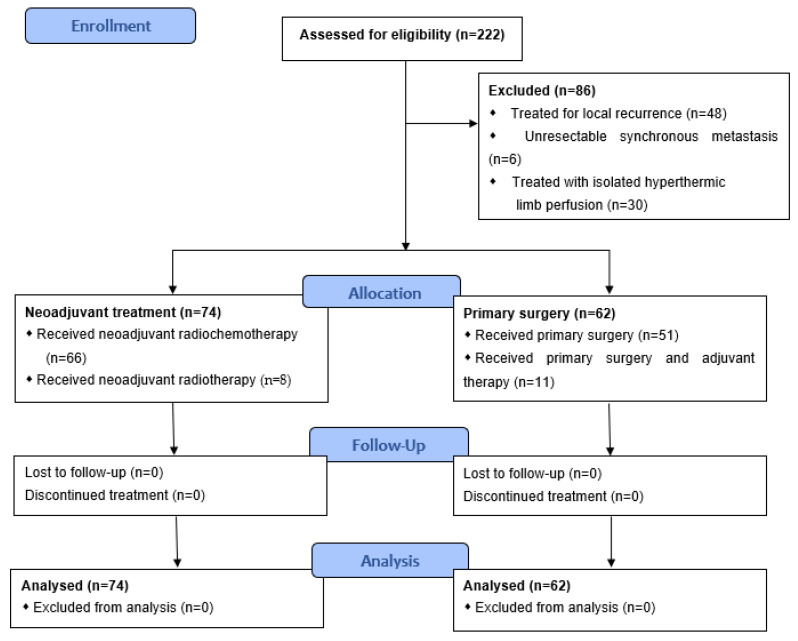
CONSORT flow diagram of the study.

All patients who were diagnosed and treated with STS of the extremities at the Department of Surgery of the University Hospital Erlangen in Germany between 2000 and 2016 were drawn from our in-house patient database. Eligible patients were those who had an age of ≥18 years and suffered from an STS of the upper or lower extremity and underwent a primary surgical treatment or neoadjuvant radio(chemo)therapy followed by surgery with curative intent at our institution. Of the 222 patients who met these criteria, 48 were excluded because they were treated for local recurrence (LR) of STS. Other exclusion criteria were patients with extended, non-resectable metastasis at time of diagnosis (*n* = 6) and patients who received isolated hyperthermic limb perfusion (ILP) in a neoadjuvant setting (*n* = 30). Two further patients were excluded, as they received both neoadjuvant and adjuvant therapy. Overall, statistical analysis was conducted with the data of 136 patients with STS (Figure 1).

A total of 101 patients (74%) were primarily referred to our sarcoma center and had a confirmation of the diagnosis by ultrasound-guided biopsy. Approximately one quarter (*n* = 35; 26%) of the patients had received an excisional biopsy confirming malignancy or a primary excision elsewhere under the false assumption of benignity resulting to incomplete tumor resection or unclear resection status. These patients were referred to our sarcoma center to further therapy. The patients of the whole collective (*n* = 136) received either PS or nRCT followed by surgery in 6 weeks.

Our analysis incorporated demographic data and clinical covariates such as age, sex, tumor location, tumor size, type of operation, histological type and grade, staging of tumor and margin status, clinical and pathological response to NT, postoperative outcome with morbidity and mortality and follow-up as well.

### 2.2. Neoadjuvant Therapy

**Figure 2:**
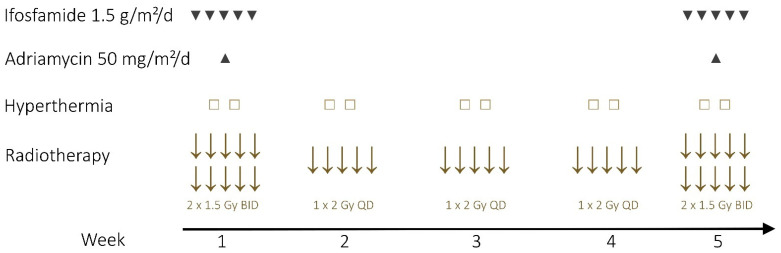
Treatment regimen based on the Erlangen schedule. (BID = twice daily RT, QD = once daily RT).

Neoadjuvant therapy was applied to patients with unresectable or borderline resectable STS of the extremities, unfavorable tumor location (infiltration of vessels, nerves and bones) and high-risk tumor (tumor size ≥ 5 cm, grade II-III, deep/extracompartmental location). Neoadjuvant therapy consisted of RT with 50 Gy to the whole tumor bearing compartment plus 10 Gy Boost to the tumor plus 4 cm with a single-dose ranging from 1 × 2 Gy to 2 × 1.5 Gy per day and-in most cases (*n* = 66))-simultaneous CTx with ifosfamide (1.5 g/m^2^/d, d1–5, q28) and doxorubicine (50 mg/m^2^/d, d3, q28) in weeks 1 and 5 (Figure 2). Eight patients could not receive or complete neoadjuvant CTx due to toxicity (*n* = 2) or patient refusal (*n* = 6). Additionally, some patients (*n* = 31/74, 42%) were treated with deep regional or superficial hyperthermia. The target temperature was 40–44 °C for 90 minutes in two sessions per week. Hyperthermia was not possible in all patients due to availability constraints.

### 2.3. Conduct of Surgery and Response Assessment

Surgical procedures included wide resection, radical/compartmental resection. Clinical response to NT was evaluated by RECIST (“Response evaluation criteria in solid tumors”) [25] and/or Choi criteria [26] as complete response (CR), partial response (PR), stable disease (SD), or progressive disease (PD). Pathological response to NT was reflected by the extent of tumor regression measured at the resection specimen. Complete remission was defined as 100% necrosis (complete absence of viable tumor cells), a near total remission was defined as 95–99% necrosis, subtotal 90–94%, partial remission with 50–89% necrosis, and stable disease with < 50% necrosis. Resection margin status was defined as R0 (macroscopically complete resection with surgical margins free of microscopic disease), R1 (macroscopically complete resection with positive microscopic surgical margins) and R2 (macroscopically incomplete resection) [27]. Staging followed the criteria of the AJCC (2017) [28]. Stage 1 and 2 were classified as low-risk STS, stage 3 and 4 as high-risk STS. Postoperative complications were classified using the Clavien-Dindo-classification [29] where post-operative complications are assigned to five groups with ascending severity.

### 2.4. Follow Up and Statistical Analysis

Follow-up parameters were measured from the date of surgery. Because the quantification of follow-up tends to be underestimated using median only, the follow-up time was estimated using the method of Kaplan–Meier estimated potential follow-up [30]. Before testing, all relevant predictors (treatment group, high-/low-risk STS, age, histological type, histological differentiation, tumor size, and surgical margins) were put in a correlation matrix and tested for multicollinearity. The classification in high-/low-risk STS and histological differentiation both had a Pearson and Spearman correlation of 0.80; thus, multicollinearity was implied for those variables. Consequently, only the classification in high-/low-risk STS was used in the following analysis.

Statistical analysis was performed with SPSS (version 21, Chicago, IL, USA). Data was analysed using Cox Proportional Hazard (PH) models [31], a semi-parametric, robust method of Gail, et al. [32], which allows simultaneous assessment of the impact of different variables on an outcome variable [33]. All hypothesizes were tested one-sided, α-error was set to 5%.

## 3. Results

### 3.1. Demographics and Clinical Characteristics

The study included 136 patients with an STS of the lower (*n* = 106; 78%) and upper (*n* = 30; 22%) extremities. Mean age was 61.8 years (Standard deviation [SD] was 15 years) with a range from 21 to 93 years. The most common histological subtypes were undifferentiated pleomorph sarcoma (25.7%), well-differentiated liposarcoma (23.5%), myxofibrosarcoma (11.8%) and leiomyosarcoma (7.4%). Those four subtypes comprised around two thirds (68.4%) of the study sample. The average tumor size at the time of diagnosis was 11.7 cm (median 10 cm, SD ±7.5 cm, range 0.5–35 cm). In 80% of all cases the STS were > 5 cm and in 25.0% larger than 15 cm. In 100 cases (73.5%) the tumors were located subfascial, whereas in 33 cases (24.3%) were epifascial in location. In total, fifty-six STS (41%) were classified as low-grade and 76 (56%) were classified as high-grade. At time of diagnosis more than half of the patients (53%) had stage I or II according to AJCC [28] and were thus classified as being low-risk, while 47% had stage III or IV (high-risk STS). The demographic and clinical characteristics are shown in Table 1.

### 3.2. Therapeutic Characteristics

Seventy-four patients (54.4%) received NT and 62 patients (45.6%) received PS. Of the group NT, the 89% of patients underwent nRCT (*n* = 66) and 11% of them (*n* = 8) underwent nRT. Of the group PS, 11 patients (11/62, 18%), who had either microscopically positive/uncertain surgical margins (3 patients with R1, one patient with RX), had turned out to be poorly differentiated (G3, *n* = 6) or had a very large (>30 cm), moderately differentiated (G2) STS (*n* = 1) received adjuvant therapy. As expected, patients with high-grade STS received significantly more often NT (χ^2^ = 34.25, *p* < 0.001) (Table 1). Interestingly, of those patients with epifascial STS (*n* = 33), NT was applied to 15 patients (45%), all of which had undergone elsewhere an incomplete/unclear resection (100%), had moderately or poorly differentiated STS (86.7% were classified as G2 (*n* = 6) or G3 (*n* = 7)) and/or unfavorable localization (53.3%).

Two thirds of the patients (67%) underwent a wide resection, while the rest (33%) underwent a compartmental resection. In 83 patients (61%), no plastic surgery was necessary, whereas 53 (39%) required plastic reconstruction. The most common types of plastic reconstruction included the use of mesh-graft-transplantations (*n* = 11), m. latissimus dorsi flaps (*n* = 13), m. rectus abdominis flap (*n* = 8) and antero-lateral-thigh flaps (*n* = 6). Postoperative complications were presented in 52 patients, with most of them (*n* = 33/52, 63%) undergoing grade I complication. The postoperative morbidity was significantly associated with the administration of NT (*n* = 37/74, 50%) compared with patients receiving PS (15/62, 24%) (*p* = 0.003). Importantly, no grade III complication was observed in the group PS, whereas eleven patients of group NT (15%) experienced grade III complications requiring interventional or surgical therapy (necrosis or abscess with wound revision (*n* = 8), lymphatic fistula formation with drain (*n* = 1), disturbed blood flow through the plastic flap (*n* = 1) and loss of split skin graft (*n* = 1). There was no postoperative mortality as one patient (0.7%) had life-threatening operation-non-related complication (cardiac arrest with successful reanimation). Of those patients with NT and grade III complications (*n* = 11), eight patients had received nRCT (8/66, 12.1%) whereas the other three patients had received only neoadjuvant radiotherapy (3/8, 37.5%). For details in therapeutic and surgical characteristics, see Table 2.

Negative resection margins (R0) were observed in 116 cases (85.3%), microscopic residual tumor in resection margins (R1) were observed in 17 cases (12.5%). There was no significant difference in the frequencies of negative resection margins in both groups (χ^2^ = 0.48, *p* = 0.487). In three patients a residual tumor could not be safely dismissed (RX).

### 3.3. Clinical and Pathological Response to the Neoadjuvant Therapy

Patients with NT had a mean tumor size of 11.3 cm (range 2.5–30.0 cm) at time of diagnosis and a mean tumor size of 9.1 cm (range 0–28.0 cm) at time of surgery. A T-downstaging was observed in 28 patients (38%). According to RECIST criteria, of the 74 patients who received neoadjuvant therapy ten patients (13.5%) showed CR. Eighteen patients (24.3%) showed a PR, whereas 31 patients (41.9%) had SD and 13 patients (17.6%) PD. According to CHOI criteria, 10 patients (13.5%) had CR. 31 patients (41.9%) showed PR, 13 patients (17.6%) had SD and 18 patients (24.3%) had PD. Overall, data of two patients were missing.

Pathological complete remission (pCR) was observed in ten cases (13%), near-total remission in 8 cases (11%), subtotal remission in 8 cases (11%) and partial remission in 34 cases (46%), whereas 14 patients (19%) showed a stable disease. Of those patients with pCR, five patients had an undifferentiated pleomorph sarcoma (50%), the rest consisted of liposarcoma (1), alveolar sarcoma (1), spindle cell sarcoma (1), fibroplastic sarcoma (1) and sarcoma NOS (1). Nine out of ten patients with a pCR had primarily undergone an incomplete tumor resection elsewhere.

### 3.4. Follow-Up

The median of Kaplan-Meier estimated potential follow-up for OS was 82 months (95%-CI: 55.31–109.27 months, range: 6–202 months). The 5-year LRFS, MFS, DFS and OS rate of the whole cohort was 89.9%, 77.0%, 70.6% and 72.6%; whereas the 5-year LRFS, MFS, DFS and OS rate was 90.5%, 67.2%, 64.1% and 62.8% for the NT group and 89.5%, 88.3%. 78.4% and 83.8% for the PS group.

Overall, 40 patients experienced a recurrence, in the form of either local failure or distant metastases, or both. Specifically, twelve patients (8.8%) developed LR during a mean follow-up of 35 months, (median 28 months, range 3–103 months). The local recurrence rate did not differ between patients with NT (*n* = 5/74; 6.8%) and PS (*n* = 7/62; 11.3%). Of those with positive surgical margins, zero patients with NT (0/8 patients) and two patients with PS (2/9 patients, 22.2%) developed LR.

Thirty-one patients (22.8%) developed distant metastases, either synchronous (*n* = 16) or metachronous (*n* = 15). The main localizations of metastases were lungs (64.5%) and lymph nodes (19.4%). Development of distant metastasis occurred more often in patients with NT than patients with PS (*p* = 0.003), because the majority of low-risk STS was included in the PS group. However, in patients with high-risk STS, occurrence of distant metastasis was lower with NT compared to PS (53.3% vs. 57.1%).

### 3.5. Prognostic Factors and Group Differences

**Figure 3:**
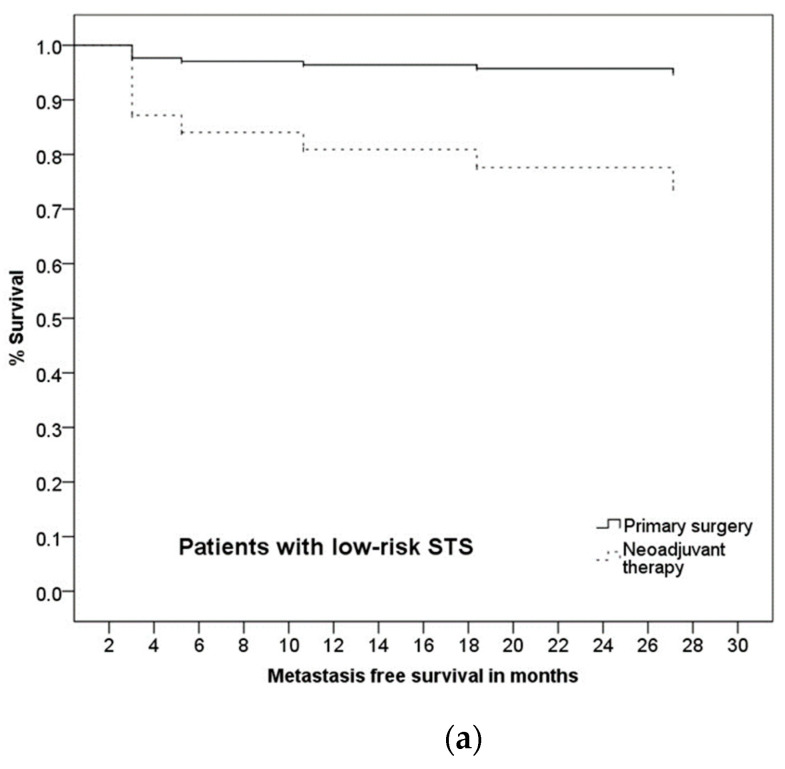

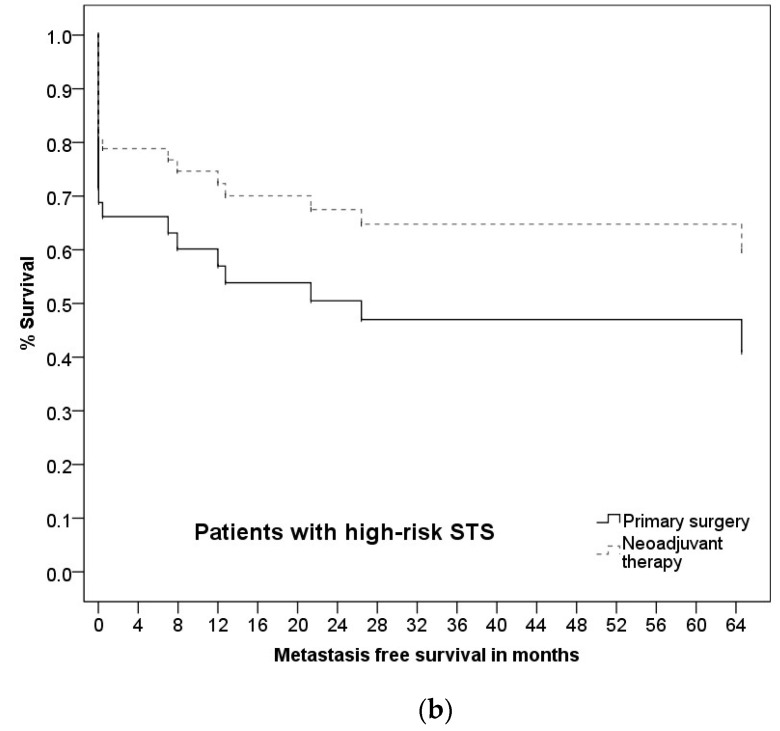
Kaplan–Meier curve of metastasis-free survival for (**a**) low-risk STS and (**b**) high-risk STS according to the therapeutic approach (PS vs. NT).

Our data did not imply any statistical survival advantage for patients who received NT in regard to OS (*p* = 0.075), LRFS (*p* = 0.569) or DFS (*p* = 0.060). Neoadjuvant therapy was a significant predictor (HR = 5.87, 95%-CI: 1.18–29.11, *p* = 0.030) of MFS, as well as the risk (high vs. low) of STS (HR = 15.83, 95%-CI: 2.60–96.48, *p* = 0.003). Interestingly, the interaction of these two variables was significant; the application of NT was associated with a better MFS in patients with a high-risk STS (HR = 0.10, 95%-CI: 0.01–0.74, *p* = 0.025, see Figure 3). This can be explained by the confounding impact of high-risk STS on developing distant metastases (HR = 15.83, 95%-CI: 2.60–96.48, *p* = 0.003). All further potential confounders (age, histological type, tumor size and surgical margins) were not significant. For details, see Table 3.

**Figure 4:**
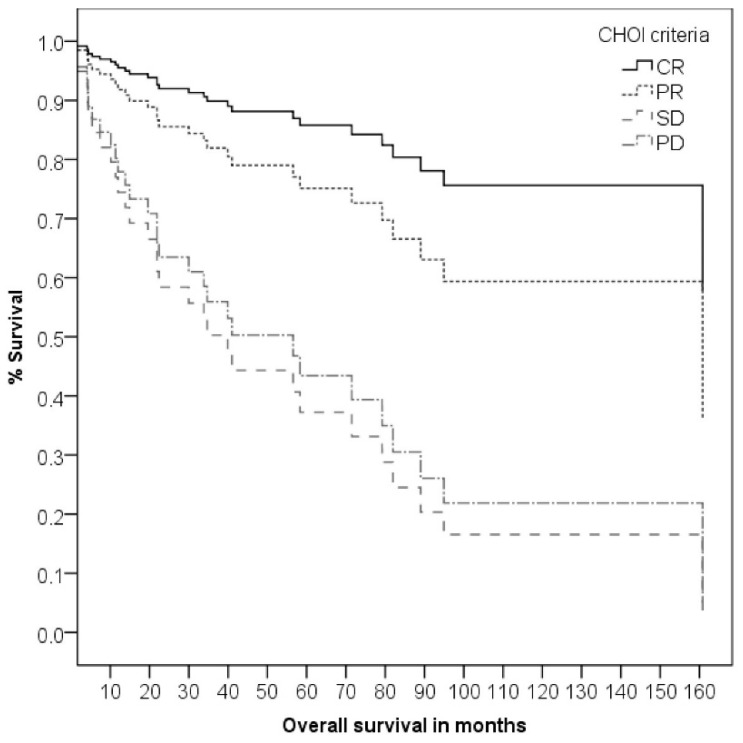
Kaplan–Meier curve of overall survival according to the CHOI-criteria.

**Figure 5:**
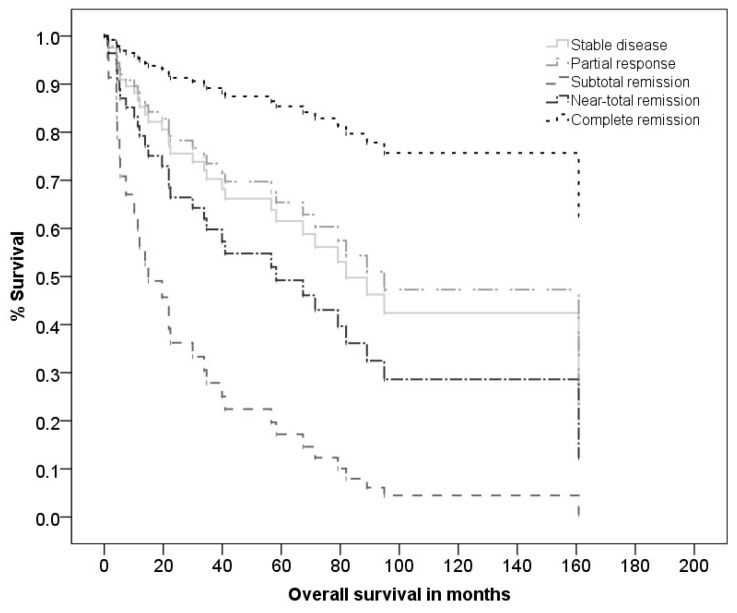
Kaplan–Meier curve of overall survival according to the histopathological response.

In contrast to RECIST criteria, CHOI criteria had a significant impact on OS (χ^2^ = 11.72, df = 3, *p* = 0.008) with SD and PD having a significantly worse OS than CR (*p* = 0.024, HR = 6.44 for SD and *p* = 0.031, HR = 5.44 for PD, see Figure 4). Pathological response rate had a significant impact on OS (χ^2^ = 12.68, df = 4, *p* = 0.013) with near-total pathological response having a significantly worse OS than CR (*p* = 0.005, HR = 11.15, see Figure 5). For the analysis of MFS, LRFS and DFS, the coefficients did not converge for both clinical and pathological response rates and are therefore not reported.

Surgical margins did not affect OS (*p* = 0.631), LRFS (*p* = 0.555), MFS (*p* = 0.871) or DFS (*p* = 0.531).

Patients with epifascial STS had a better OS (*p* = 0.047), MFS (*p* = 0.037) and DFS (*p* = 0.011) than patients with subfacial STS. However, LRFS was not affected by tumor depths (*p* = 0.101). The administration of NT in patients with epifascial STS did not offer any advantage regarding OS (*p* = 0.318), LRFS (*p* = 0.358), MFS (*p* = 0.419) or DFS (*p* = 0.829) in comparison to PS.

**Figure 6:**
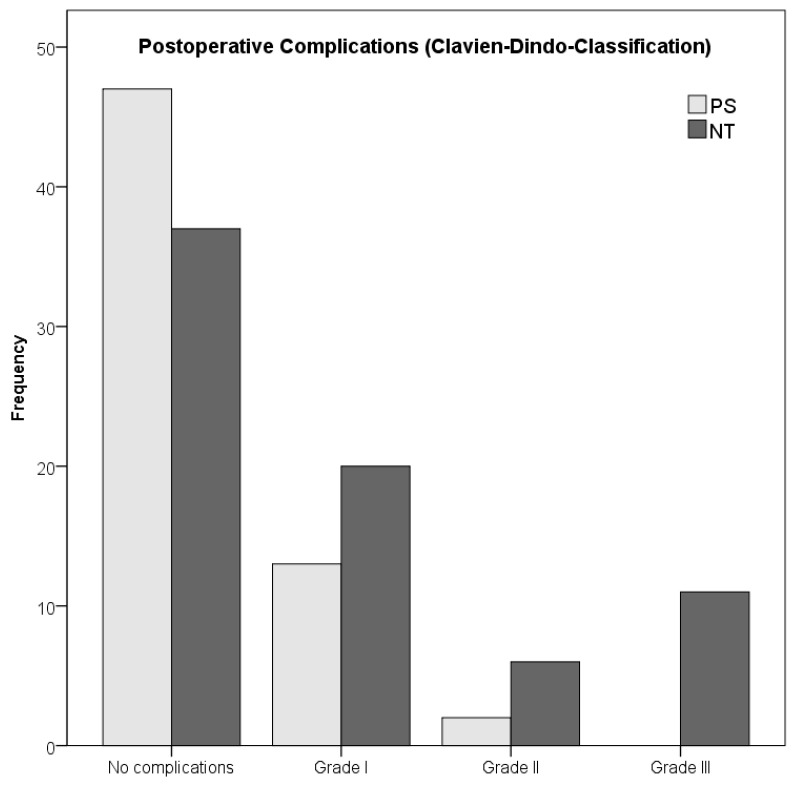
Development of postoperative complications with and without NT.

The chances to develop postoperative complications was 3.13-fold higher when receiving NT in comparison to PS (Fishers exact test, χ^2^ = 15.91, *p* = 0.001, Cramérs V = 0.33, OR = 3.13, see Figure 6).

## 4. Discussion

The treatment of STS of the extremities is a challenging clinical problem due to the rarity and heterogeneity of these tumor entities. The introduction of neoadjuvant treatment modalities, consisting of radiotherapy, chemotherapy, targeted therapy, radiosensitizers or their combination, is nowadays considered an equal or even better therapeutic option to adjuvant therapy. Particularly, neoadjuvant therapy has attracted increasing attention in patients with locally advanced disease due to several advantages in comparison to adjuvant therapies (e.g., tumor devitalisation, lower radiation dose, smaller irradiated volume, shorter treatment time, lower later toxicity) [34]. Until now, few studies have examined the effects of a combined nRCT. One of the largest studies by Mahmoud et al. [15] provided some evidence that combined nRCT may be better than surgery only and better than RT or CTx alone. It was showed that implementation of RT and CTx in high-grade sarcomas reduced the risk of death by 37% and 24%, retrospectively, compared to surgery alone. Five-year-OS rate was 35.7% for surgery only, 49.9% for either RT or CTx, and 62.1% for combined RCT. However, no difference in regard to chronological sequence of adjunct therapy (neo- or adjuvant) was observed [15]. Interestingly, Chowdhary et al. [35] analyzed a total of 884 high-risk STS patients with STS of the extremities and/or trunk and demonstrated that the addition of neoadjuvant CTx to neoadjuvant RT led to an improved OS. Other authors provided similar rates of LRFS and OS between RT and RCT groups [36]. There is however some evidence that anthracycline- and ifosfamide-based nRCT yield advantages over the single application of nRT *or* CTx [11,12,37,38]. Especially the regimen used at our institution has been evaluated with some promising results in the past [16,17]. In comparison to other studies, our treatment regimen differs from the often used preoperative 50 Gy in 1.8–2.0 Gy fractions [39] providing 60 Gy as opposed to the more common 50 Gy. However, the preoperative commonly used 50 Gy in 1.8–2.0 fractions are not based upon robust evaluation and should be reevaluated [40]. In the present study, our findings support the notion that high-risk STS profit from nRCT in terms of treating microscopic metastatic disease or reducing the risk for development of metastasis, which could be attributed to the addition of CTx to nRT. Although the other differences in survival between our two groups (NT vs. PS) were not significant, we provided excellent oncological outcomes and survival rates, especially very low LR rates (8.8% for the whole cohort with 11.3% for PS and 6.8% for NT).

The clinical and pathological response to neoadjuvant therapy may influence or possibly predict outcomes. Ten patients (13.5%) in our study showed a pCR, half of which had an undifferentiated pleomorph sarcoma. None of these patients had a local recurrence or occurrence of distant metastasis during the observed period. This result is compatible with findings of other authors who found a pCR rate of 8.3% (4/48) [11] and 21.9% (14/64) [14], who treated patients with nRCT (MAID regimen, 44 Gy). Because there has been no available data about the sarcoma subtypes showing a pCR and there have been conflicting results regarding the prognostic value of pathologic response (necrosis) after NT [41,42,43,44], further research is needed to evaluate these parameters or even additional factors like hyalinization or fibrosis [45,46].

NT was associated with a 3.13-fold greater risk of postoperative complications in comparison to PS. A total of 11 patients (8.1%) with NT had major (grade III) complications. Overall, the rate of severe postoperative complications is thus lower than the rates of other authors [18,19,21,24,47,48], though most of those studies only examined the effects of nRT. Götzl et al. [47] also observed statistically significant differences in major complication rates after nRCT vs. no RT (28 vs. 7%, *p*  <  0.001). Tseng et al. [24] and Rosenberg et al. [48] found that 31% of patents with nRT had severe postoperative complications, Cannon et al. [18] found 34%, Cheng et al. [19] found 31%, O’Sullivan et al. [21] found 35% and Davis et al. [20] found 31.5%, although a direct comparison is flawed due to different definitions of ‘severe postoperative complications’ and due to the use of nRT instead of nRCT. In our study, eight patients with NT and grade III complications had nRCT (8/66, 12.1%), whereas three patients had nRT (3/8, 37.5%). Although the numbers are too small for reliable statistical analysis, this provides evidence that nRT alone might have a higher rate of postoperative complications and that the addition of nCTx is a safe option that might even reduce the risk for postoperative complications.

Besides, NT was applied to 15 patients with epifascial STS, who all underwent an incomplete tumor resection elsewhere. Patients with epifascial STS and NT did not differ in regard to OS, LRFS, MFS or DFS compared to patients with PS. The reason for NT was usually incomplete outpatient excision (100%), poor histological differentiation (86.7% were classified as G2 or G3) and/or unfavorable localization (53.3%). Therefore, it might be beneficial to apply NT to patients with epifascial STS under less-than-optimal circumstances. To our knowledge, there have not been any studies regarding this aspect. Therefore, further research is needed.

Remarkably, there was no significant difference between patients with R0- and patients with R1-resection in regard to LRFS in our study population. This is in contradiction to the findings of Mack et al. [9], who did find a significant negative impact of positive surgical margins (present in 10.7% of the patients, and marginal margins in 44%) on LRFS in univariate analysis. Negative impact of positive surgical margins on LRFS has been also observed in other studies with mostly high rate of positive margins [15,49,50,51,52]. On the other hand, Sadoski et al. [53] and Gronchi et al. [42] did not find any significant impact of positive surgical margins in patients with nRT on the LR (who had positive margins [R1] in 21.2% and 9.5% of cases, respectively). Our study demonstrated a low rate of positive surgical margins, which does not allow any reliable statistical analysis regarding the impact of margins on survival, whereas not finding significance between the two groups might be due to the overall very low number of LR (8.8% for the whole group with 11.3% for PS and 6.8% for NT).

Due to the rarity of STS retrospective comparison is often used to analyze treatment effectiveness, but one has to keep in mind that the allocation into different treatment groups did not happen at random: Locally advanced cases of high-grade STS tended to receive NT while patients with low-grade STS tended to undergo PS, which might have led to unobserved confounding influences (for example due to unobserved complications in regard to CTx or other underlying diseases), making it thus more difficult to analyze the true effect of NT. Furthermore, our study is a single-center study and is therefore prone to uncertainty regarding the transfer to other contexts. For example, only 8.8% of our study population developed a LR, which is comparatively rare. To secure external validity and to get a bigger sample size, more multicenter studies should be conveyed; an analysis via hierarchic linear models could hereby control for institutional effects. The development of a better and closer network in the research of STS seems to be without alternative. In Germany, some steps towards that goal have already been taken [54].

## 5. Conclusions

In conclusion, our study provided important insight into the effectiveness of NT in patients with STS. We could show that multimodal treatment strategies lead to excellent oncological outcomes in patients with STS of the extremities. The addition of neoadjuvant CTx to nRT leads to better oncological outcomes in patients with high-risk STS regarding MFS and is associated with a low number of postoperative complications. Further research in the form of prospective study designs is warranted.

## Figures and Tables

**Table 1:** Demographic, clinical and histopathological characteristics of patients (*n* = 136).

	nRT (*n* = 74)	PS (*n* = 62)	Total (*n* = 136)	*p*-Value **
Sex, no. (%)				
Male	43 (58.1%)	30 (48.4%)	73 (53.7%)	0.302 a
Female	31 (41.9%)	32 (51.6%)	63 (46.3%)	
Age in years				
Mean (±SD)	61.69 (±13.57)	61.89(±16.61)	61.8 (±15.0)	0.940 b
Median	64.0	63.5	64.0	
Range (min.-max.)	24–87	21–93	21–93	
Histological Type, no. (%)				
Undifferentiated pleomorph sarcoma	28 (37.8%)	7 (11.3%)	35 (25.7%)	<0.001 a
Well differentiated Liposarcoma	3 (4.1%)	29 (46.8%)	32 (23.5%)	<0.001 c
Myxofibrosarcoma	8 (10.9%)	8 (12.9%)	16 (11.8%)	0.706 a
Leiomyosarcoma	5 (6.7%)	5 (8.1%)	10 (7.4%)	0.771 a
Myxoid liposarcoma	5 (6.7%)	4 (6.5%)	9 (6.7%)	1.000 c
Synovialsarcoma	5 (6.7%)	2 (3.2%)	7 (5.1%)	0.454 c
Spindle cell sarcoma	5 (6.7%)	1 (1.6%)	6 (4.4%)	0.219 c
Dedifferentiated liposarcoma	3 (4.1%)	0 (0%)	3 (2.2%)	0.250 c
Angiosarcoma	0 (0%)	2 (3.2%)	2 (1.5%)	0.206 c
Alveolar sarcoma	1 (1.4%)	1 (1.6%)	2 (1.5%)	1.000 c
Other sarcoma *	11 (14.9%)	3 (4.8%)	14 (10.3%)	0.087 c
Tumor location, no. (%)				
Upper Extremity	15 (20.2%)	15 (24.2%)	30 (22%)	0.679 a
Lower Extremity	59 (79.8%)	47 (75.8%)	106 (78%)	
Tumor depth, no. (%)				
Epifascial Tumor	15 (20.2%)	18 (29.1%)	33 (24.3%)	0.248 a
Subfascial Tumor	57 (77.1%)	43 (69.3%)	100 (73.5%)	
Missing value	2 (2.7%)	1 (1.6%)	3 (2.2%)	
Initial tumorsize in cm				
Mean (±SD)	11.3 (±6.3)	12.0 (±8.8)	11.6 (±7.5)	0.617 b
Median	10.0	9.8	10.0	
Range (min.-max.)	2.5–30.0	0.5–35.0	0.5–35	
G-Grading (histological differentiation)				
G1	4 (5.4%)	33 (53.3%)	37 (27.2%)	<0.001 a
G2	20 (27.0%)	14 (22.6%)	34 (25.0%)	
G3	45 (60.8%)	10 (16.1%)	55 (40.4%)	
G4	1 (1.4%)	1 (1.6%)	2 (1.5%)	
GX	3 (4.0%)	3 (4.8%)	6 (4.4%)	
Missing value	1 (1.4%)	1 (1.6%)	2 (1.5%)	
High-/low grade STS				
Low-grade	14 (18.9%)	42 (67.7%)	56 (42.4%)	<0.001 a
High-grade	58 (78.4%)	18 (29.1%)	76 (57.6%)	
Missing value	2 (2.7%)	2 (3.2%)	4 (2.9%)	

* this included fibrosarcoma (1), fibromyxoid sarcoma (1), sclerosing epitheloid sarcoma (1), extraskeletal osteosarcoma (1), myxoid chondrosarcoma (1), rhabdomyosarcoma (1), fibroplastic sarcoma (1), myofibroblastic sarcoma (1), myofibroplastic sarcoma (1), pleomorph myxoid sarcoma (1), pleomorph myogene sarcoma (1) and sarcoma NOS (3). ** a = Chi^2^-Test, b = T-Test, c = Fishers Exact Test.

**Table 2:** Therapeutic and surgical characteristics (*n* = 136).

	NT (*n* = 74)	PS (*n* = 62)	Total (*n* = 136)	*p*-Value *
Group, no. (%)				
Primary surgery	--	62 (100%)	62 (45.6%)	--
Neoadjuvant therapy	74 (100%)	--	74 (54.4%)	--
RCT	66 (89.2%)	--	66 (48.5%)	
With hyperthermia	29	--		
RT	8 (10.8%)	--	8 (5.9%)	
With hyperthermia	2			
Adjuvant therapy	--	11 (17.7%)	11 (8.1%)	
RCT	--	6 (9.6%)	6 (4.4%)	
RT	--	4 (6.5%)	4 (2.9%)	
CTx	--	1 (1.6%)	1 (0.7%)	
Outpatient practice primary R1-excision, no. (%)				
No outpatient practice primary excision	53 (71.5%)	48 (77.5%)	101 (74.3%)	0.441
Outpatient practice primary excision	21 (28.5%)	14 (22.5%)	35 (25.7%)	
Surgical procedure, no. (%)				
Wide resection	46 (62.2%)	45 (72.5%)	91 (66.9%)	0.198
Compartmental resection	28 (37.8%)	17 (27.5%)	45 (33.1%)	
R-Stage (residual tumor)				
R0	65 (87.8%)	51 (82.3%)	116 (85.3%)	0.482
R1	8 (10.8%)	9 (14.5%)	17 (12.5%)	
RX	1 (1.4%)	2 (3.2%)	3 (2.2%)	
Postoperative complications, no. (%)				
No complications	37 (50.0%)	47 (75.8%)	83 (61.0%)	0.003
With Complications	37 (50.0%)	15 (24.2%)	52 (38.2%)	
Grade I	20 (27.0%)	13 (20.9%)	33 (24.3%)	
Grade II	6 (8.1%)	2 (3.3%)	8 (5.9%)	
Grade III	11 (14.9%)	0 (0%)	11 (8.1%)	
Grade IV	0 (0%)	0 (0%)	0 (0%)	
Grade V	0 (0%)	0 (0%)	0 (0%)	

* Chi^2^-Test.

**Table 3:** Kaplan-Meier means (±SE) for high-/low-risk STS in patients with NT (*n* = 74).

Survival Rates	Low-Risk STS (*n* = 24)	High-Risk STS (*n* = 46)	*p*-Value *
OS	111.97 (±10.00)	92.93 (±13.22)	0.018 *
LRFS	127.01 (±7.64)	176.19 (±9.32)	0.962
MFS	104.91 (±11.83)	120.79 (±14.51)	0.346
DFS	99.21 (±12.46)	115.65 (±14.76)	0.442

* log-rank test.

## Data Availability

The data that support the findings of this study are available from the corresponding author upon reasonable request.
